# Supplementary figures and images for: Transcription reshapes RNA hairpin folding pathways revealed by all-atom molecular dynamics simulations
Source: PLoS Comput Biol. 2025 Sep 8;21(9):e1013472. doi: 10.1371/journal.pcbi.1013472 (PMC12431647; doi:10.1371/journal.pcbi.1013472)

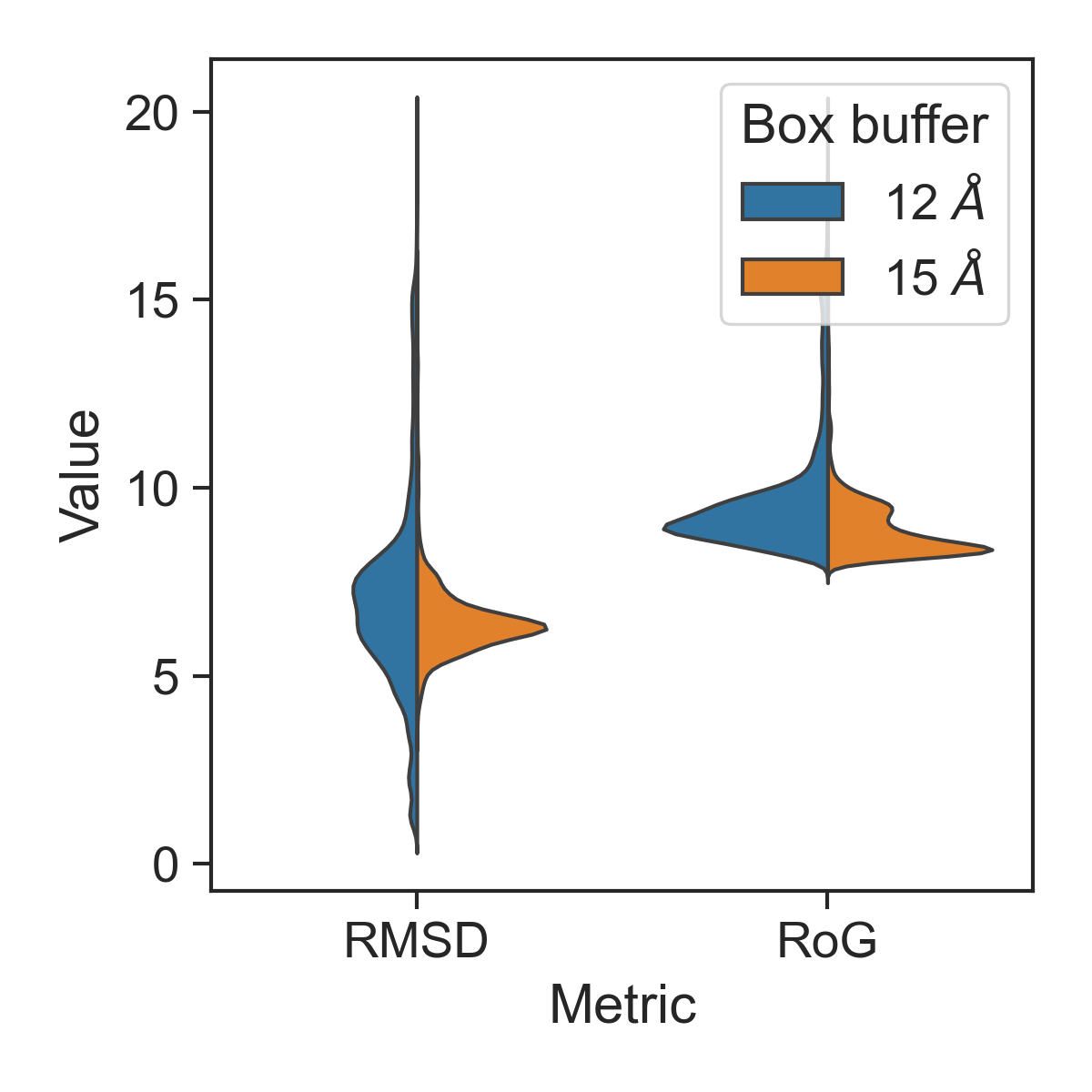

Supplement: S1 Fig — (TIF) [file pcbi.1013472.s001.tif]

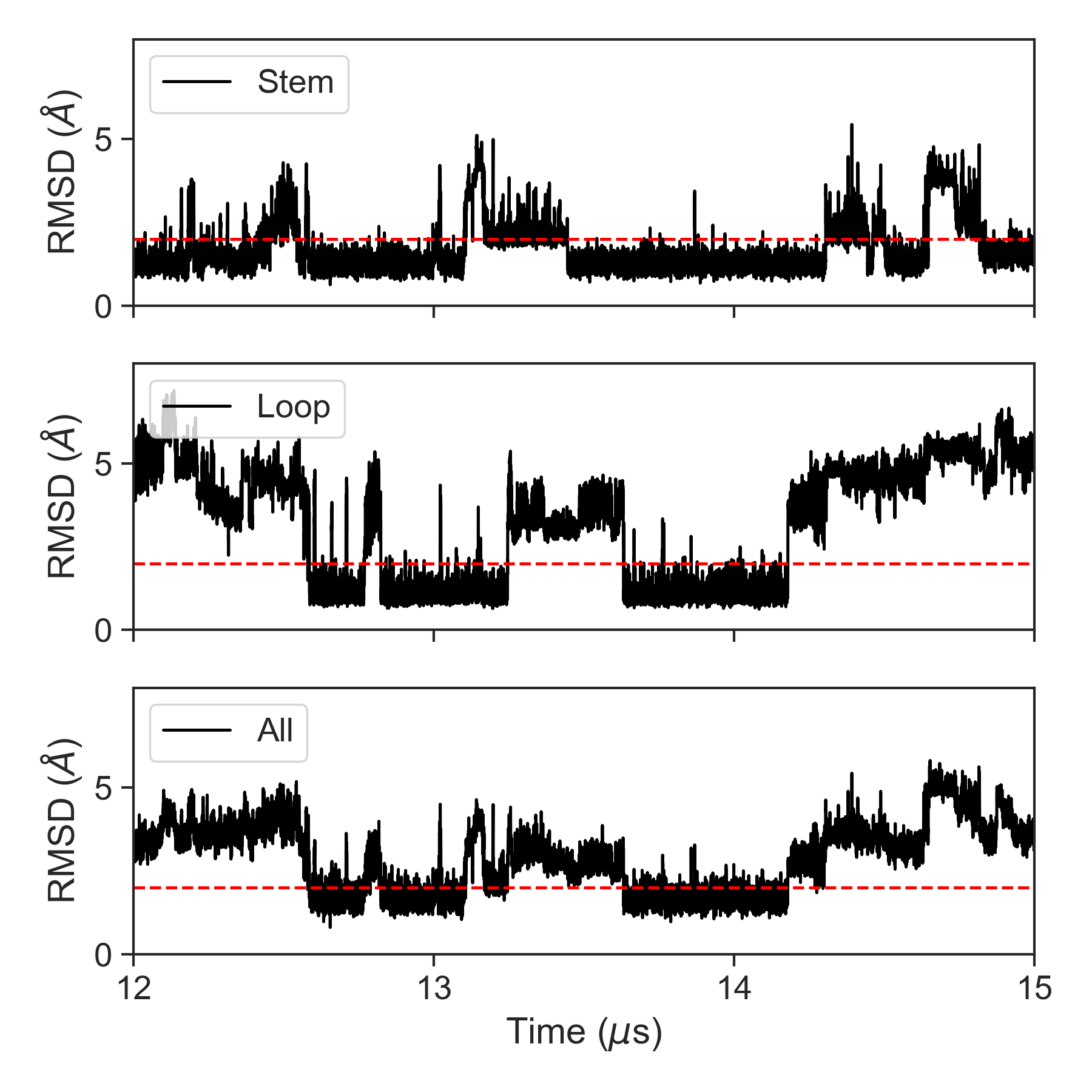

Supplement: S2 Fig — (TIF) [file pcbi.1013472.s002.tif]

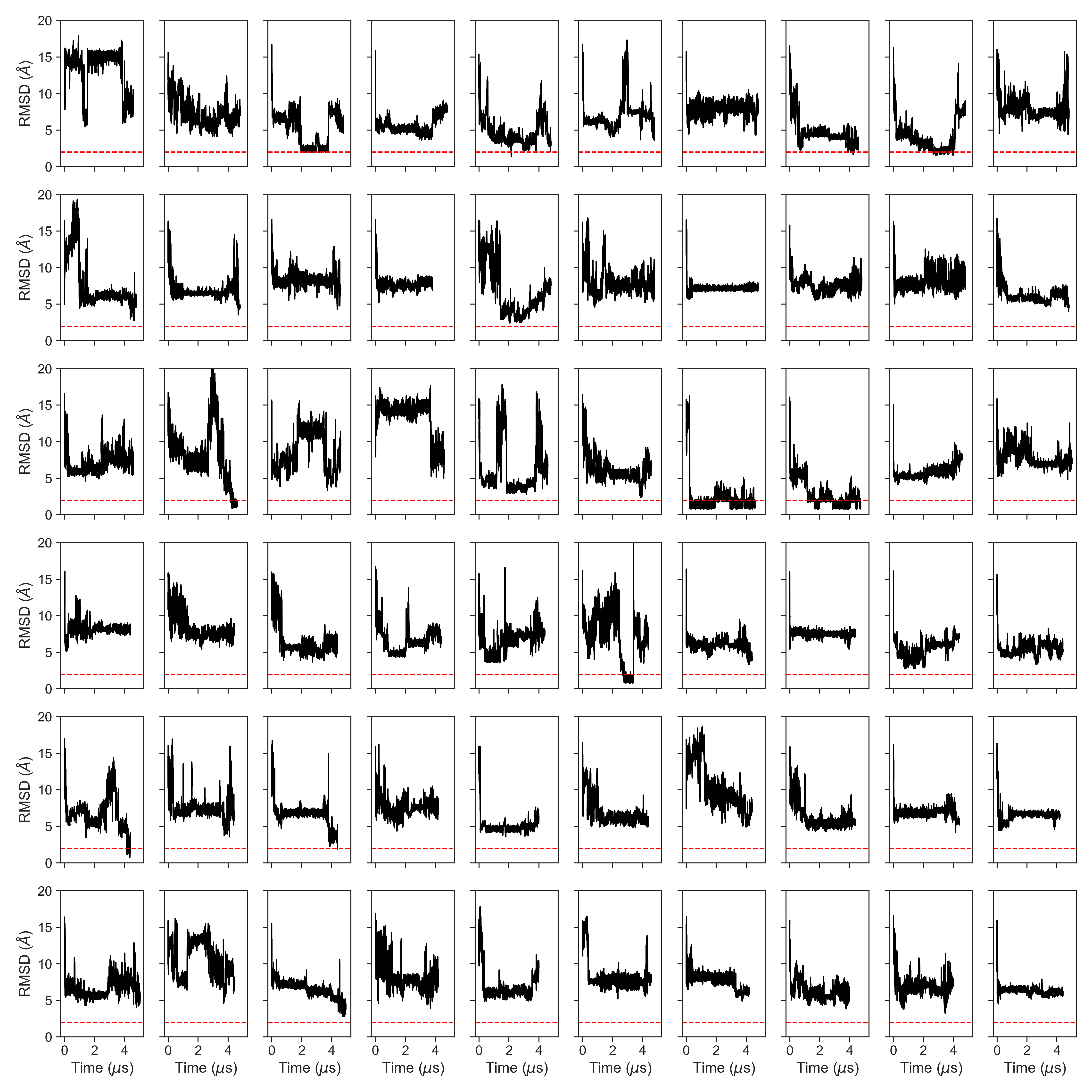

Supplement: S3 Fig — (TIF) [file pcbi.1013472.s003.tif]

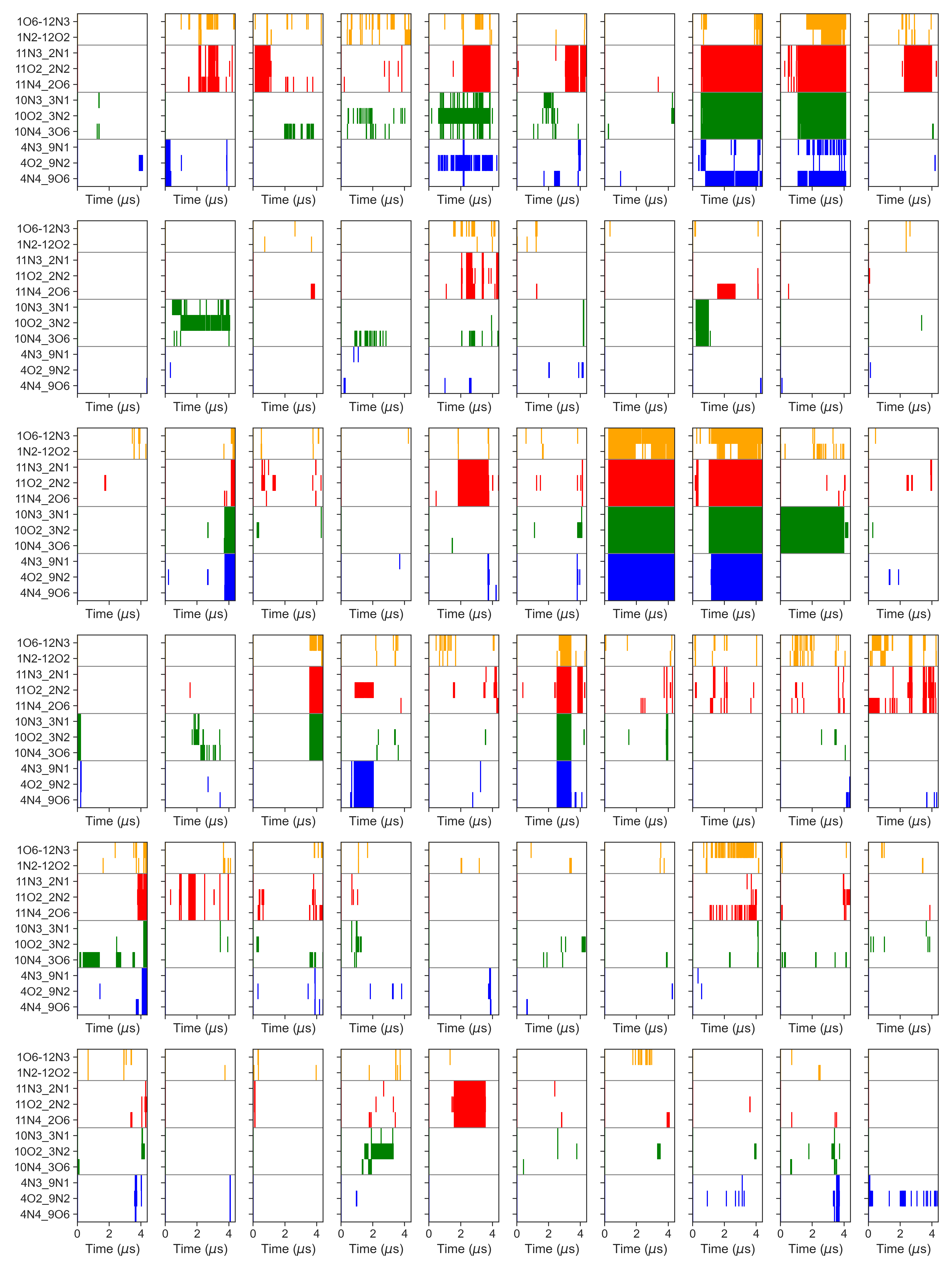

Supplement: S4 Fig — (TIF) [file pcbi.1013472.s004.tif]

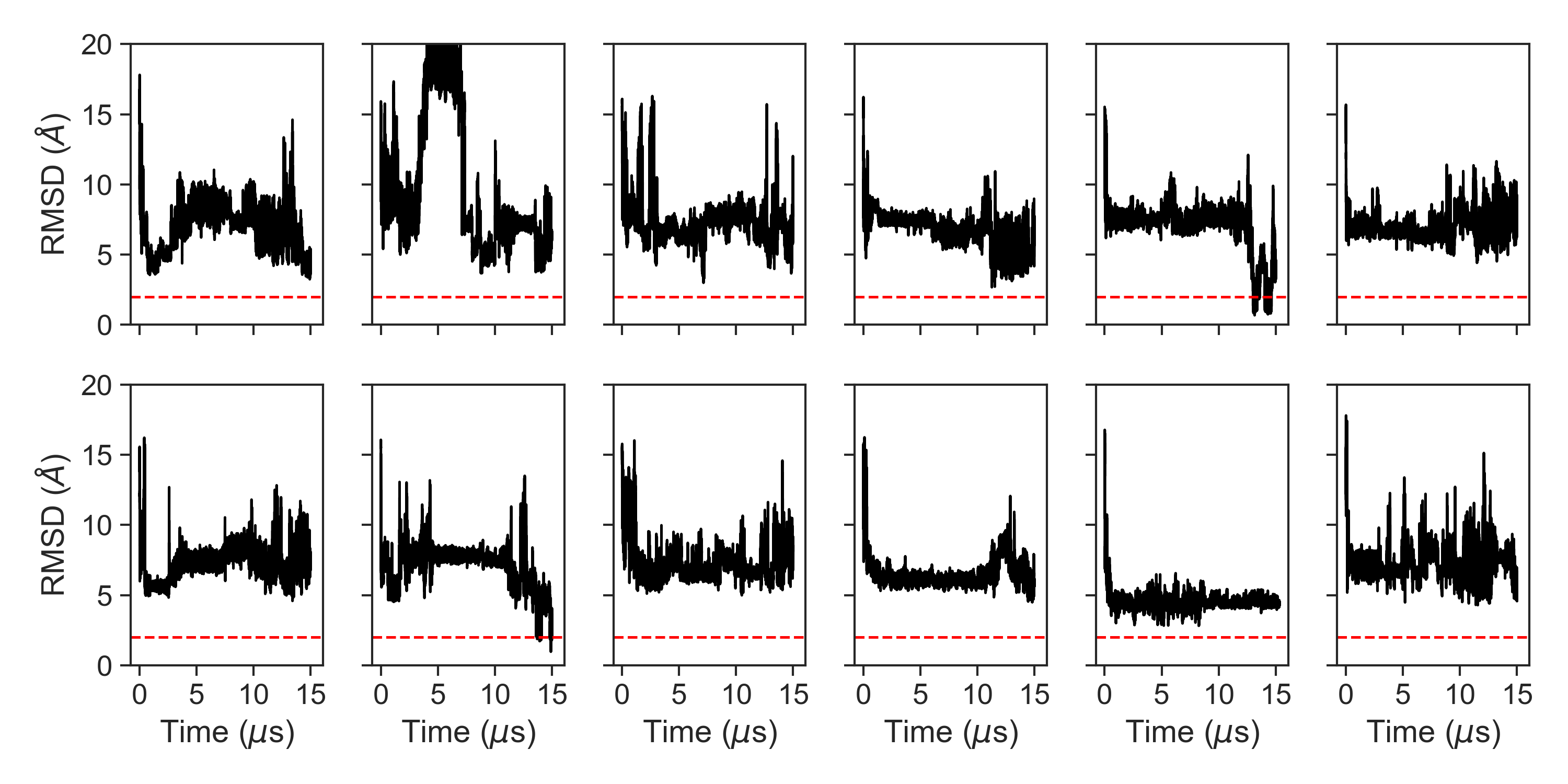

Supplement: S5 Fig — (TIF) [file pcbi.1013472.s005.tif]

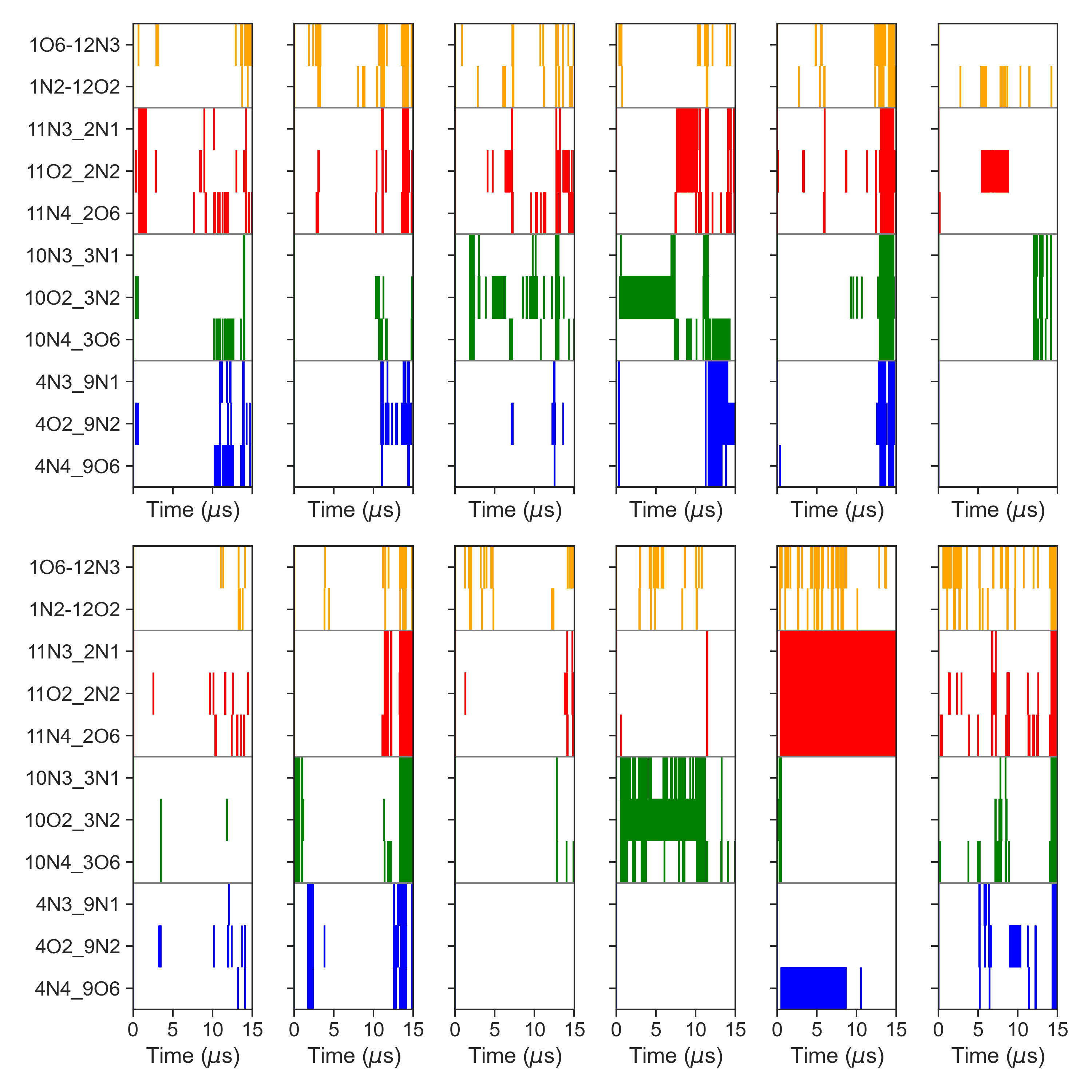

Supplement: S6 Fig — (TIF) [file pcbi.1013472.s006.tif]

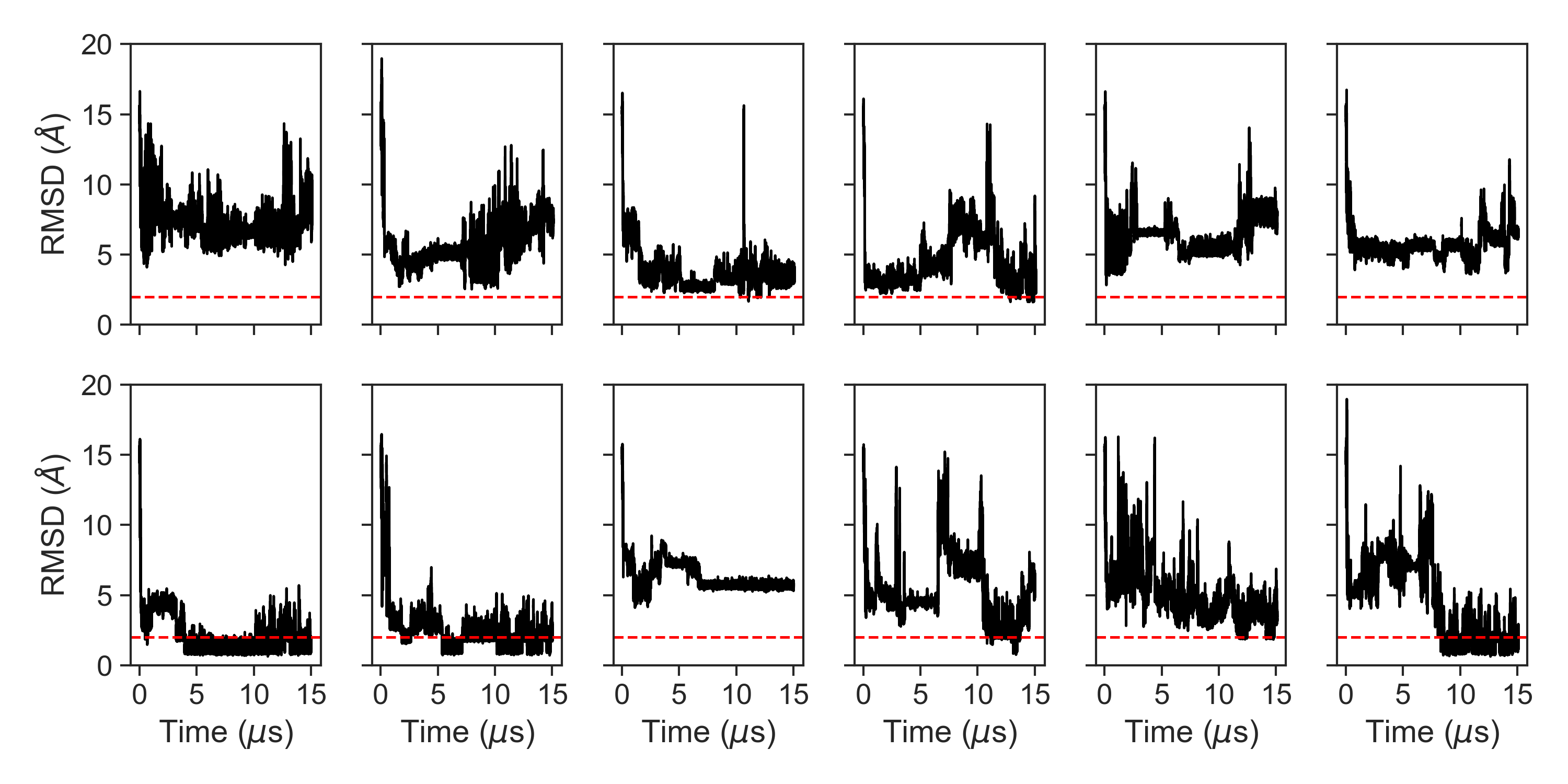

Supplement: S7 Fig — (TIF) [file pcbi.1013472.s007.tif]

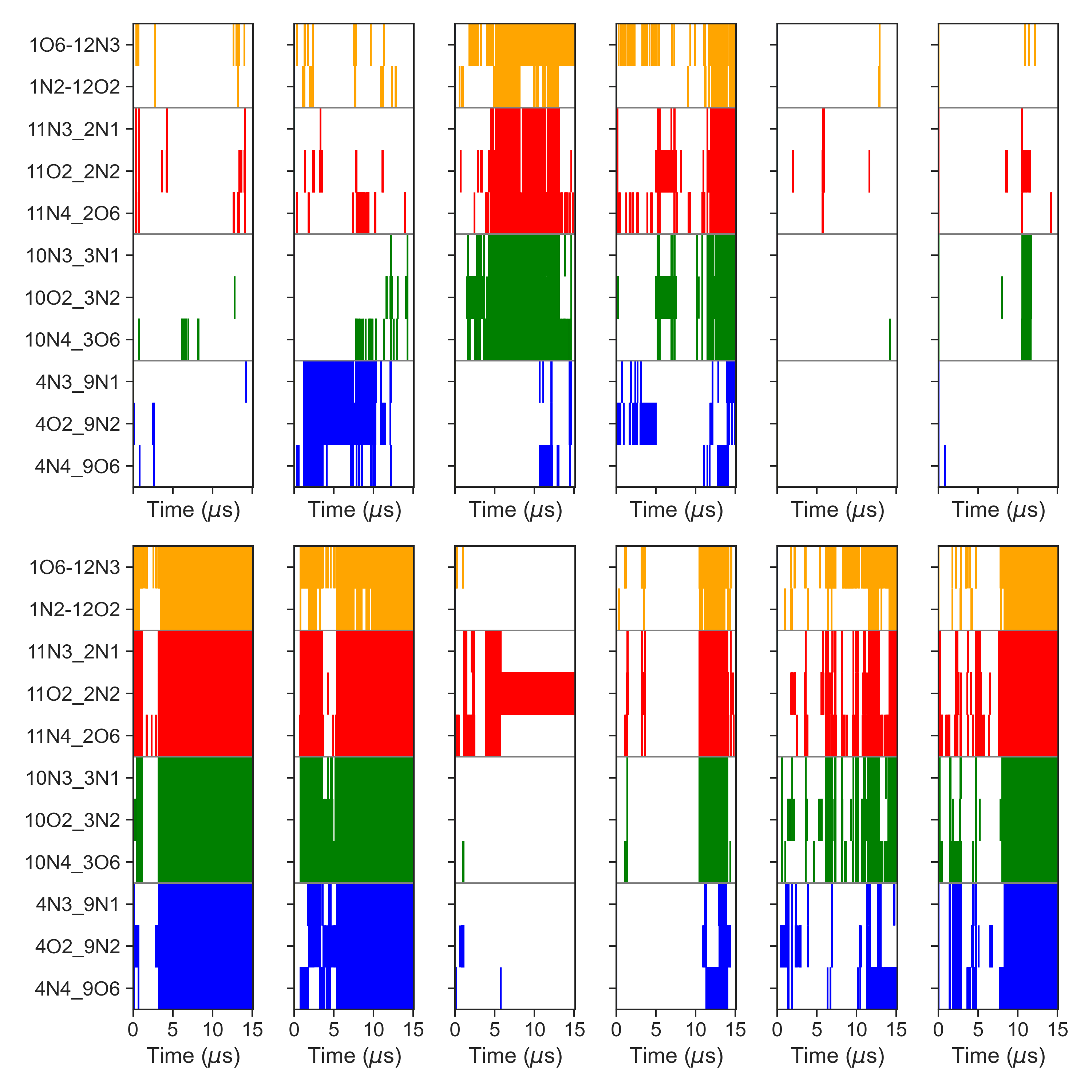

Supplement: S8 Fig — (TIF) [file pcbi.1013472.s008.tif]

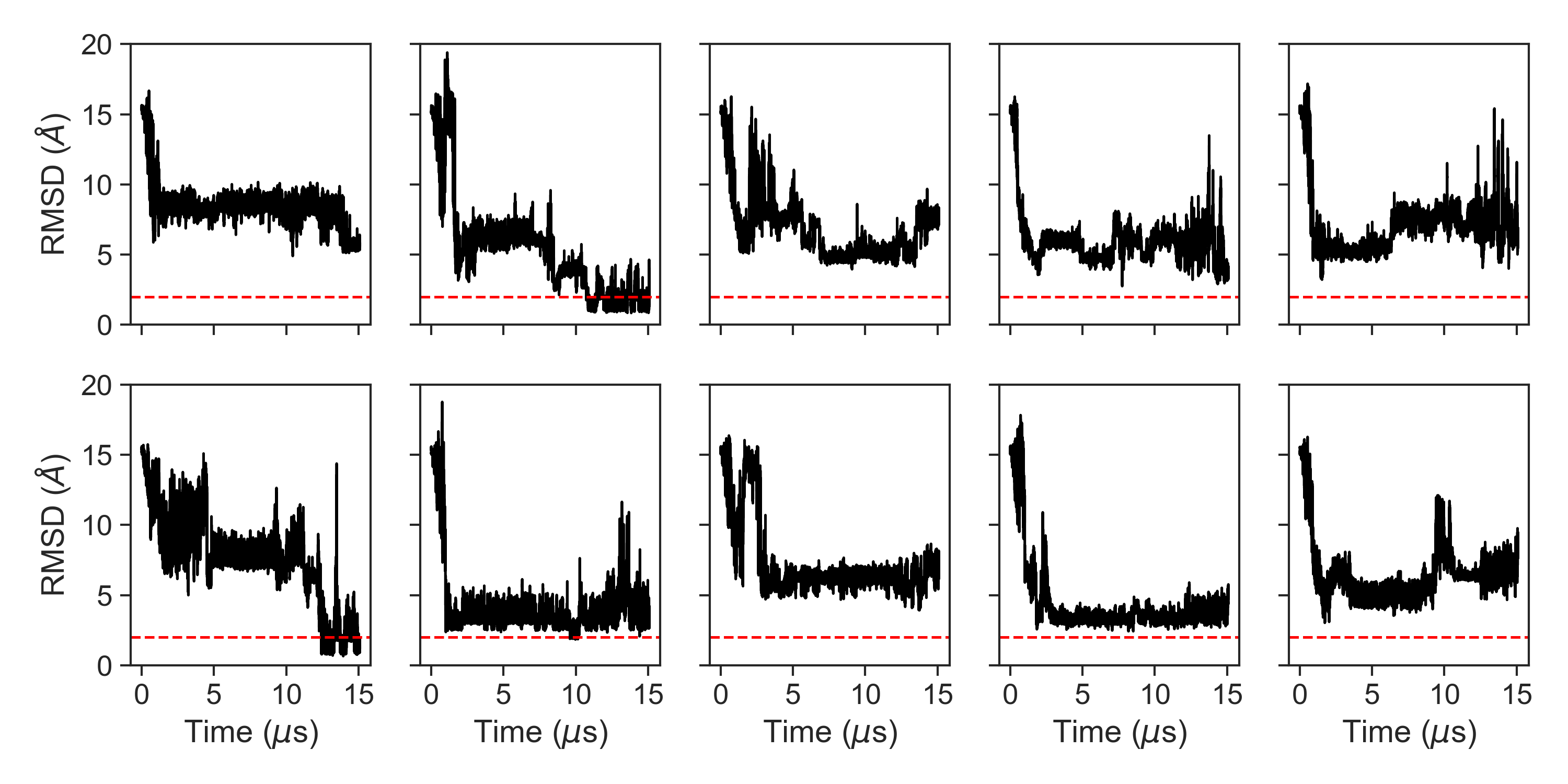

Supplement: S9 Fig — (TIF) [file pcbi.1013472.s009.tif]

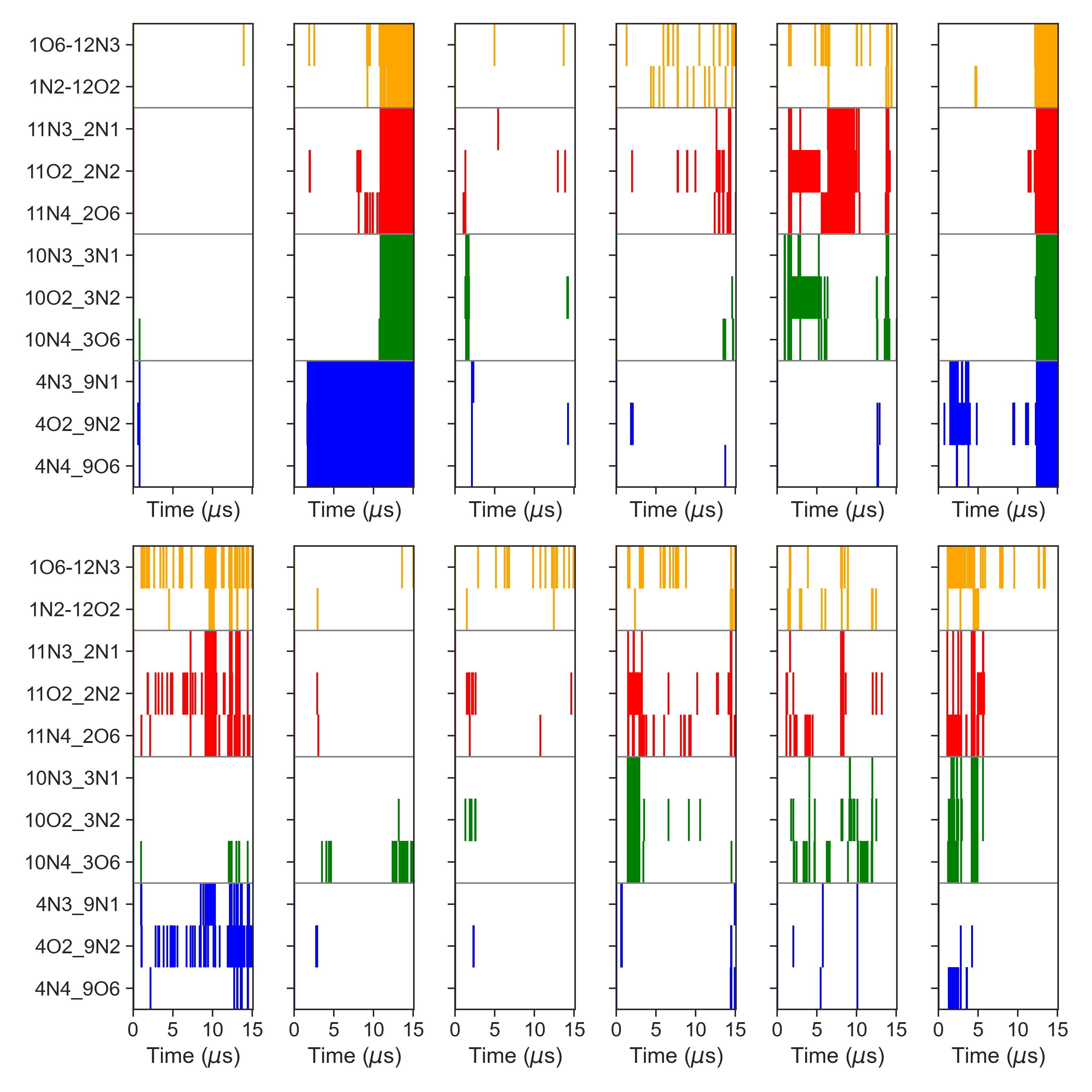

Supplement: S10 Fig — (TIF) [file pcbi.1013472.s010.tif]

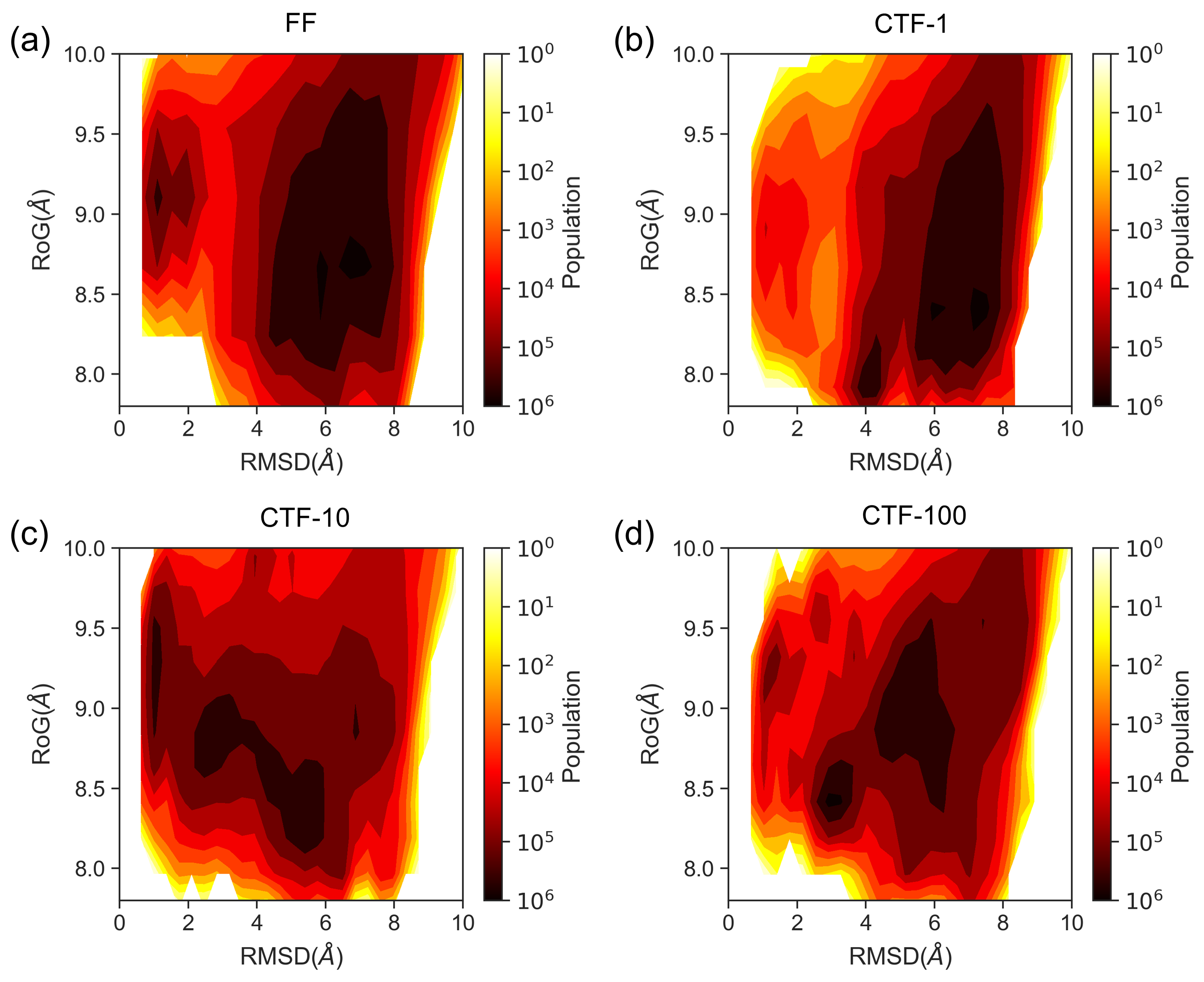

Supplement: S11 Fig — (TIF) [file pcbi.1013472.s011.tif]

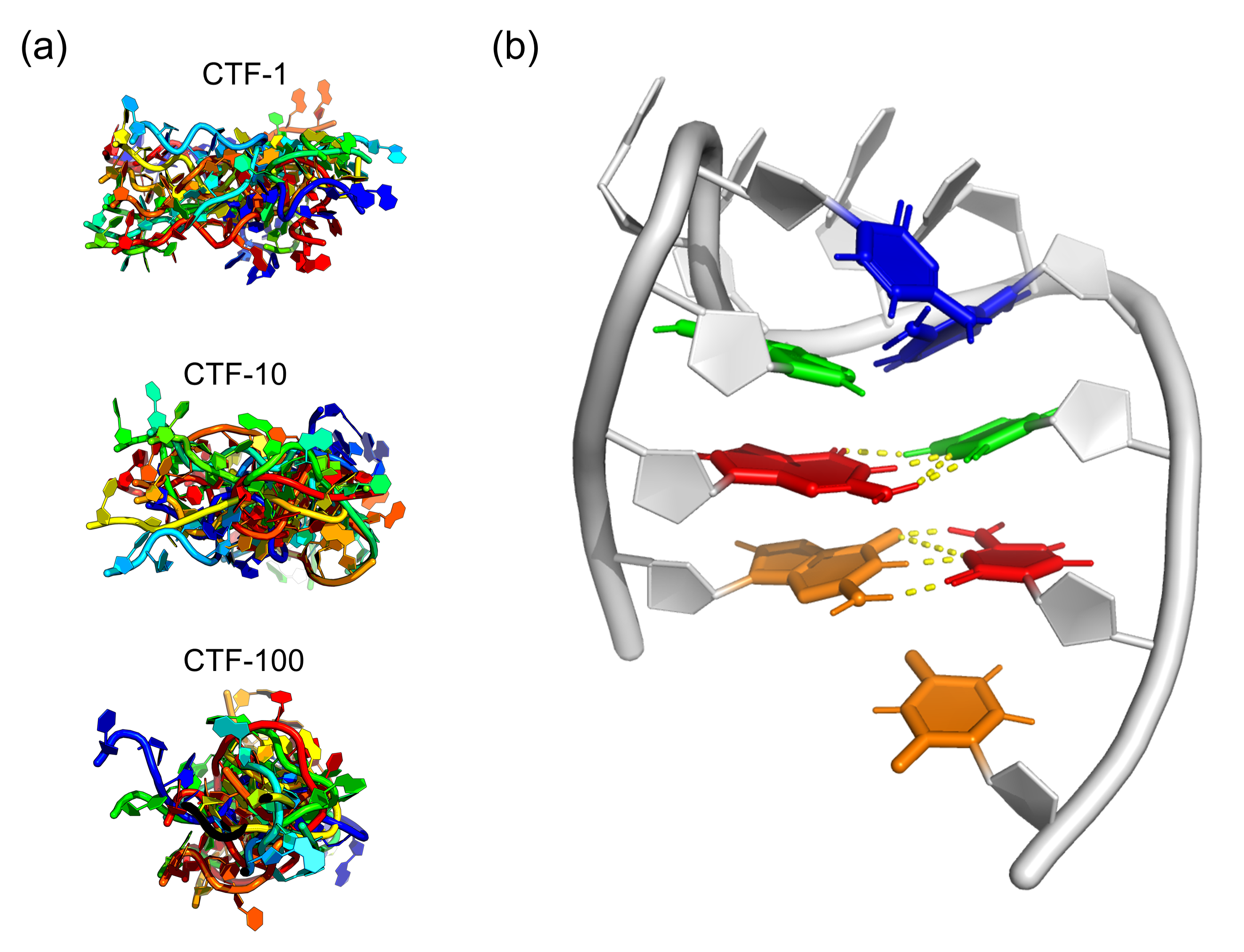

Supplement: S12 Fig — (TIF) [file pcbi.1013472.s012.tif]

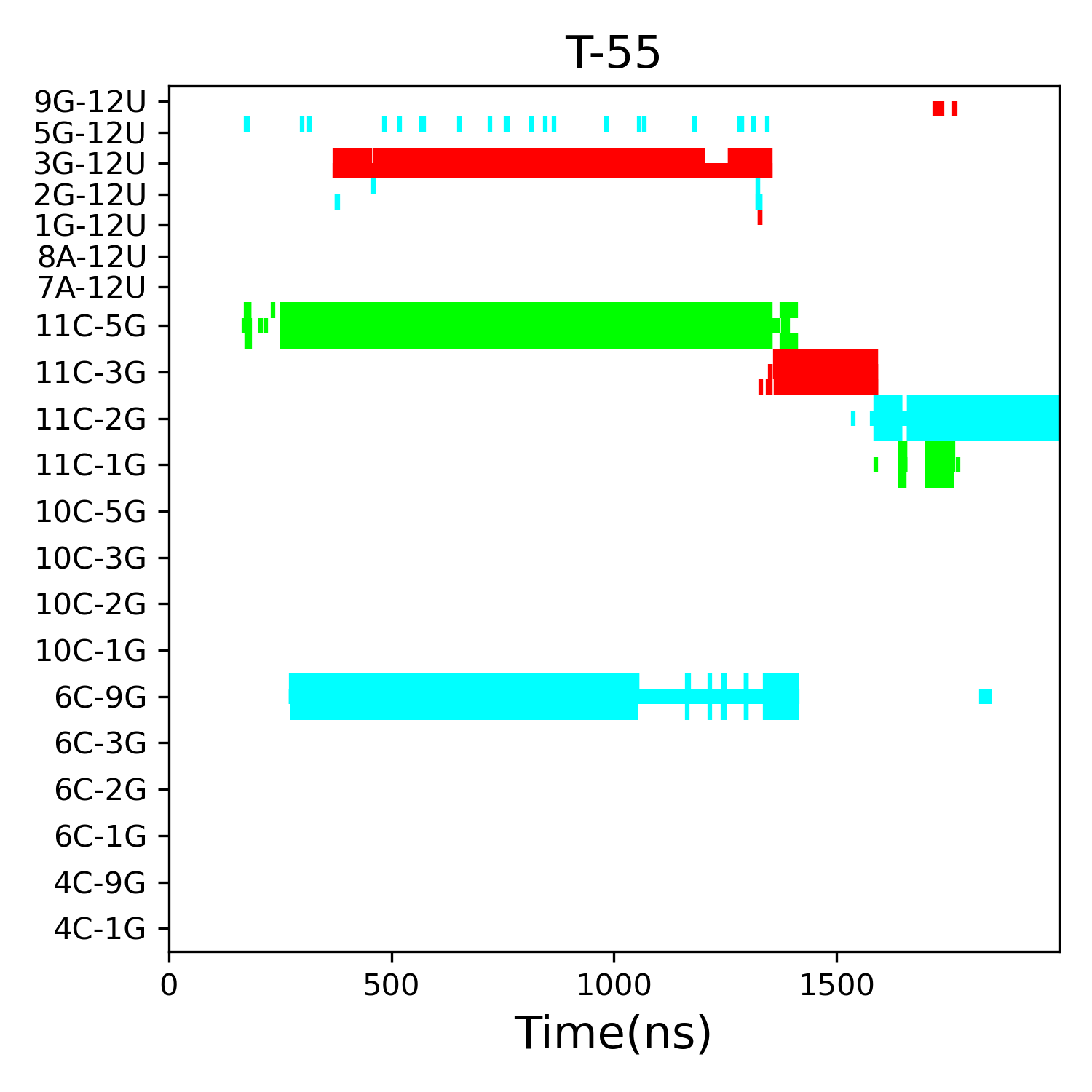

Supplement: S13 Fig — (TIF) [file pcbi.1013472.s013.tif]
