# Supplementary material for: Transcription reshapes RNA hairpin folding pathways revealed by all-atom molecular dynamics simulations
Source: PLoS Comput Biol. 2025 Sep 8;21(9):e1013472. doi: 10.1371/journal.pcbi.1013472 (PMC12431647; doi:10.1371/journal.pcbi.1013472)
Supplement: Attachment — Submitted filename: response.docx [file pcbi.1013472.s015.docx]

**-----------------**

**Reviewer #1**

**-----------------**

RNA folding during transcription is a complex process, collectively influenced by many factors, such as the speed of elongation, site-specific pausing and interactions between the nascent RNA chain and the RNA polymerase. This manuscript by Tao et. al. introduces a simplified all-atom molecular dynamic framework for RNA cotranscriptional folding simulations. Although it focuses on the exploration of transcription speed in RNA hairpin cotranscriptional folding, the method it provides for atomic-scale mechanistic studies provides a new approach for understanding the intricate dynamics of RNA folding. This study is intriguing, however, there are still several inquiries that need to be addressed.

**Response:** We thank you for the positive assessment of our work and the constructive feedback. In this revised manuscript, we have carefully addressed each of the issues raised and made corresponding revisions. We hope our detailed responses and modifications are satisfactory.

(1) RNA free folding (FF) begins with a completely unstructured coil, while RNA cotranscriptional folding (CTF) involves a chain growth process where the RNA folds as it elongates. By the time the full chain is synthesized, its structure is not fixed, but it typically differs from the coil structure. The subsequent folding process represents a refolding of the entire chain. Thus, the distinction between FF and CTF can be understood as a difference in initial structure. In the article, the authors chose different simulation times for FF and CTF (3-4 μs vs. 15 μs). It is advisable to sample FF for the same duration as CTF, while also noting that this 15 μs should not include the chain elongation time. This adjustment would ensure a more reasonable comparison between the two folding processes.

**Response:** We thank you for this insightful suggestion. We agree that comparing FF and CTF simulations using different sampling durations could indeed introduce bias, as CTF can be considered a specific type of FF starting from a different initial structure.

In the original study, to investigate the CTF mechanism of 1ZIH, we simulated 10 independent CTF trajectories per transcription speed, each 15 μs long. The 60 FF trajectories, however, were partially inherited from our previous study (Duan et al., Phys Rev E 102, 032403 (2020)) focusing on base-pair formation; consequently, these initial FF trajectories were only 1–2 μs each. To utilize existing data, we previously extended each FF trajectory by an additional 2 μs. While this resulted in shorter individual trajectories for FF (3–4 μs) compared to CTF (15 μs), the total simulation time was somewhat balanced (~200 μs for FF vs. ~150 μs per CTF condition), partially mitigating the disparity.

In the revised manuscript, we took substantial steps to further reduce this discrepancy and enhance statistical robustness. For FF, we extended each of the 60 existing trajectories by an additional 1 μs, resulting in individual trajectory lengths of 4–5 μs (total FF sampling: 271.9 μs). For CTF, we added two new, independent ~15 μs trajectories per transcription speed, resulting in 12 trajectories per speed (total CTF sampling per speed: > 180 μs). These add > 150 μs of new simulation data. Notably, extending all 60 FF trajectories to 15 μs and increasing CTF trajectories to 60 per speed would require an estimated compute time exceeding 5 years on our current resources (8 × RTX 3080 Ti GPUs, generating ~2 μs aggregate data per day). Therefore, the implemented strategy represents the optimal and feasible compromise achievable within the 3-month revision timeframe (the editor initially granted two months, and we applied an additional one-month extension).

The updated data have been fully analyzed and are presented in the revised manuscript. The enhanced sampling yielded new productive folding events (Fig. 4b): one additional PATH I and one PATH II trajectory for FF; one PATH II event each for CTF-1 and CTF-10. These findings strengthen our revised conclusion that FF preferentially folds along PATH I, while CTF exhibits a strong bias towards PATH II.

**Fig. 4. Folding pathways of 1ZIH.**

**Supplementary Fig. S3. Temporal evolution of RMSD in 60 FF simulations.**

**Supplementary Fig. S4. Temporal evolution of 11 hydrogen bonds in 60 FF simulations.**

**Supplementary Fig. S5. Temporal evolution of RMSD in 12 CTF-1 simulations.**

**Supplementary Fig. S6. Temporal evolution of 11 hydrogen bonds in 12 CTF-1 simulations.**

**Supplementary Fig. S7. Temporal evolution of RMSD in 12 CTF-10 simulations.**

**Supplementary Fig. S8. Temporal evolution of 11 hydrogen bonds in 12 CTF-10 simulations.**

**Supplementary Fig. S9. Temporal evolution of RMSD in 12 CTF-100 simulations.**

**Supplementary Fig. S10. Temporal evolution of 11 hydrogen bonds in 12 CTF-100 simulations.**

(2) FF has 60 independent simulations, while each transcription speed for CTF only provides 10. It is advisable to increase the number of CTF simulations. This would enhance the statistical significance of the different folding pathways, allowing for a more robust comparison and better understanding.

**Response:** We thank you for highlighting this important point. To enhance the statistical power of our pathway analysis, we have increased the number of independent CTF trajectories for each transcription speed from 10 to 12 in the revised manuscript. This expansion strengthens the robustness of our comparative analysis between CTF and FF and provides greater confidence in the observed pathway preferences.

(3) Figure 5 is very important, but it needs to include the cases for CTF-1 and CTF-10. Additionally, the discussion in Section 3.3 regarding the impact of transcription speed on CTF folding is not sufficiently thorough. It would also be beneficial to add annotations for the initial structures (coil for FF and post-transcription structures for CTF) in Fig. 5. This would enhance the ability to observe how transcription speed influences the initial conformations and folding processes.

**Response:** We thank you for these constructive comments. In the revised manuscript we have expanded Fig. 5 to include the CTF-1 and CTF-10 data, following the suggestion of Reviewer #2 to restrict the analysis to trajectories that successfully reach the native state; the corresponding distributions based on all simulated trajectories are now provided as Supplementary Fig. S11.

As shown in the new Fig. 5, CTF conformations sampled across all three transcription speeds populate regions of smaller RMSD and RoG compared to FF. Since the intermediates of PATH II are inherently more compact than those of PATH I, these regions likely represent PATH-II intermediates, thereby rationalizing the preferential folding of CTF along PATH II. The same conclusion also holds when all trajectories are included (Supplementary Fig. S11).

Given that CTF can be conceptualized as FF initiating from a specialized initial structure, we systematically examined post-transcription conformations. Fig. 6a displays the conformational distributions at T_F_ = 0 ns (the free folding time, defined as the time elapsed after transcription completion) for all trajectories. Crucially, slower transcription yields more pronounced differences between CTF and FF distributions at T_F_ = 0 ns. Tracking conformational evolution at later timepoints (T_F_ = 10, 100, and 1000 ns; Figs. 6b-d), we observe that while differences between FF and CTF distributions diminish over time, the discrepancy still persists. Additionally, we found that at T_F_ = 0 ns, the slower the transcription speed, the greater the difference between the CTF and FF conformation distributions. At later times (i.e., T_F_ > 10 ns), the CTF-10 ensemble displays the smallest RMSD and RoG values. This heightened compactness correlates with CTF-10 achieving the highest folding success rate (4 out of 12 trajectories reaching the native state), suggesting that transcription speed exerts a non-monotonic influence on folding efficiency.

Collectively, these results elucidate a pathway regulation mechanism for CTF: Transcription generates compact initial conformations rarely sampled in FF. Consequently, CTF establishes a distinct preference for folding pathways during the subsequent post-transcriptional folding compared to FF, and this pathway preference is modulated by transcription speed.

This mechanism finds compelling parallel in protein biosynthesis: Till et al. (PNAS 122, e2422678122, 2025) demonstrated that the ribosome and Trigger Factor collaboratively compact nascent proteins to accelerate folding. Despite differences between biological systems (protein vs. RNA), these analogous findings strongly support our proposed pathway regulation mechanism in CTF.

**Fig. 5. Conformational distributions in 1ZIH simulations.** (a) Conformational distribution in free folding (FF) simulations. The reaction coordinates for the conformational space are the root-mean-square deviation (RMSD) of the stem region and the radius of gyration (RoG) of the entire 1ZIH molecule. (b-d) Conformational distribution in co-transcriptional folding (CTF) simulations at a transcription speed of 1, 10 and 100 ns/nt. The conformational distributions are built only on trajectory data from successful folding.

**Supplementary Fig. S11. Conformational distributions in 1ZIH simulations.** (a) Conformational distribution in free folding (FF) simulations. The reaction coordinates for the conformational space are the root-mean-square deviation (RMSD) of the stem region and the radius of gyration (RoG) of the entire 1ZIH molecule. (b-d) Conformational distribution in co-transcriptional folding (CTF) simulations at a transcription speed of 1, 10 and 100 ns/nt. The conformational distributions are built on all simulated trajectories.

**Fig. 6. Conformational evolution of 1ZIH.** (a–d) Conformational distributions of 1ZIH under four simulation scenarios (FF, CTF-1, CTF-10, and CTF-100) at different free-folding times (T_F_ = 0, 10, 100, and 1000 ns). In FF simulations T_F_ equals the actual simulation time, whereas in CTF simulations T_F_ is obtained by subtracting the transcription duration (11Δt) from the actual simulation time. The reaction coordinates describing the conformational space are the root-mean-square deviation (RMSD) of the stem region and the radius of gyration (RoG) of the entire 1ZIH molecule. Small semi-transparent symbols denote the conformations of 1ZIH at T_F_; red squares, green triangles, blue stars, and purple circles correspond to the FF, CTF-1, CTF-10, and CTF-100 simulations, respectively. Large opaque symbols represent the means of each group, with error bars indicating the standard deviations.

Additional minors:
(4) On p6 “Systems were solvated in TIP3P (12 Å buffer)”: A buffer of this size may be limited, as it might not adequately accommodate the structural changes and dynamics during the folding process.

**Response:** Thank you for raising this potential concern. In our study, the water box dimensions with a 12 Å buffer are [81.1Å × 47.1Å × 56.2Å]. Since the average RoG of 1ZIH during simulations is below 10 Å, this box size is sufficient in most scenarios. To explicitly address this concern, we performed a 10-μs validation simulation of free folding trajectory using a 15 Å buffer. The results demonstrate similar RMSD and RoG distributions under both buffer conditions (see Supplementary Fig. S1), confirming that the 12 Å buffer is appropriate for this study.

**Supplementary Fig. S1. Comparison of the distribution of RMSD and RoG using different water buffer sizes.**

(5) On p10 “The criteria for hydrogen bond formation were based on a maximum donor-acceptor distance of 3.5 Å and a minimum donor-proton-acceptor angle of 120°.” It would be beneficial to include references that support these criteria.

**Response:** We thank you for the suggestion. The hydrogen bond criteria correspond to the default geometric parameters in CPPTRAJ, the trajectory analysis tool in Amber. These thresholds are widely adopted in molecular dynamics studies. We have added the primary reference for CPPTRAJ to this section: “Roe and Cheatham, PTRAJ and CPPTRAJ: Software for Processing and Analysis of Molecular Dynamics Trajectory Data, Journal of Chemical Theory and Computation, 9 (2013) 3084-3095.”

(6) On p11, the reference to “see Supplementary Fig. S2 for the others” should be corrected to Fig. S5.

**Response:** Thank you for identifying this error. We have corrected the reference in the revised manuscript.

(7) On p13, “with slower transcription rates favoring PATH II emergence” seems inconsistent with the data presented in Fig. 4b, where CTF-1, which has the fastest transcription rate, favors PATH II. Additionally, FF can be considered a special case of cotranscriptional folding with an infinitely fast rate, yet it favors PATH I. Authors need to clarify these observations.

**Response:** We thank you for highlighting this inconsistency in our original phrasing. The revised manuscript clarifies that faster transcription rates empirically favor PATH II emergence, as demonstrated in Fig. 4b where CTF-1 (fastest rate) exhibits the strongest bias toward PATH II. Conversely, FF—which can be conceptualized as CTF with an infinitely fast transcription rate—shows a preference for PATH I. There are two possible hypotheses to reconcile these observations:

1. Sampling limitations: Our current simulations captured an insufficient number of productive folding events for certain conditions. For example, CTF-100 (slowest rate) yielded only two successful folding trajectories, precluding robust statistical analysis of transcription rate effects across the full spectrum (Fig. 4b).
2. Non-monotonic impact of transcription rate: The relationship between transcription rate and folding pathway preference may not be linear. We hypothesize an evolutionarily optimized critical transcription rate that maximizes folding efficiency. Deviations from this optimal rate may alter pathway preferences or reduce folding efficiency.

These points are now explicitly discussed in our revised manuscript.

(8) The term "inaccessible" is too absolute. It would be more accurate to state that the structures and folding pathways sampled by CTF are also accessible to FF; however, the probabilities of sampling these structures and pathways differ.

**Response:** Thank you for this comment. As you anticipated, PATH II is not entirely inaccessible during FF folding. After extending FF simulation time to enhance conformational sampling, we observed one trajectory successfully folding along PATH II. This confirms that the structural ensembles and folding pathways sampled by CTF are also accessible to FF, though their sampling probabilities differ due to transcription-induced conformational biasing. The original absolute description has been revised accordingly.

**-----------------**

**Reviewer #2**

**-----------------**

While this manuscript was interesting, engaging to read, deliberate in its approach to investigate FF vs. CTF, I find that it is preliminary and makes several assumptions that are either testable by further analysis of the current dataset, or testable by additional simulations. I really hope the authors refine this work, as I would be really interested to read a much deeper analysis of their simulation set, as would the rest of the RNA dynamics community.

**Response:** We sincerely appreciate your constructive comments and suggestions. In this revised version, we have incorporated the necessary changes to address your comments. We hope that our revisions meet with your approval.

The authors introduce a bias in the form of a rigid plane to mimic the behavior of nucleotides directly after transcription. No interactions are allowed between the nucleotides on either side of the plane. This is an elegant solution to the problem of modeling this with “accessory” proteins, but I’m not sure it’s strictly correct – GNRA TLs aren’t necessarily formed in a single 12mer transcript, but as part of larger structures. The authors should discuss the implications of this.

**Response:** We appreciate your insightful critique regarding the use of a rigid plane to mimic nascent transcript constraints. We agree that GNRA tetraloops (TLs) *in vivo* typically fold within larger structural contexts (e.g., riboswitches or ribozymes), where flanking sequences may influence folding kinetics. We have pointed out this issue in the revised manuscript and will employ more complete transcripts in our future work.

Using only the stem as a measure of “folded” is incorrect here – the 2 Angstrom RMSD cutoff for “folded” is a particularly low bar considering one wouldn’t need to actually fold the loop correctly at all to achieve it. I am concerned that the graphical abstract is misleading – do the 2 or 3 simulations which “fold” (i.e., attain the 11 h-bonds in the stem) ever display the correct loop conformation? If not, having an arrow pointing to the “native” fold is incorrect. If they do attain the correct loop fold, you should explain that as part of the manuscript.

**Response:** We thank you for this critical insight regarding our folding criteria. We agree that relying solely on stem formation (attaining 11 h-bonds and/or RMSD ≤ 2.0 Å) is insufficient to define native folding, as it neglects the loop conformation—which is functionally essential for GNRA tetraloops. However, we did not adopt a strict definition of folding (i.e., simultaneous formation of the stem and loop), mainly because although we simulated more than 800 us of all-atom folding trajectories, only 14 trajectories successfully folded the stem. If we further required successful folding of the loop, the number of strictly successful folding trajectories would be reduced to 5 (a representative example is shown in Supplementary Fig. S2), making subsequent analysis impossible. Based on your comments, we have added the above explanation in the revised manuscript.

**Supplementary Fig. S2. An example of a completely folded trajectory.** The three rows from top to bottom show the changes in RMSD values for Stem, Loop, and All (Stem+Loop), respectively. The red dashed line represents the threshold for 2Å.

The sampling for each situation/case was investigated extremely well.
I appreciate the detail in h-bonding criteria in the Methods section.
Figure 1 is very nice and I like that all the colors are consistent there and throughout the text.

**Response:** We are deeply grateful for your kind comments.

As written, Figure 4B Table title is confusing. This table could be clearer by adding the number out of the total number of trajectories sampled (3/60). If you have only sampled each path once, then the results are different than if you mean that Path I is the only path that is sampled, and it is sampled in each trajectory.

**Response:** We thank you for identifying the ambiguity. We agree with your point and have made the following changes in Fig. 4b.

**Fig. 4.** **Folding pathways of 1ZIH.**

Page 13, line 12: “These results establish that transcription-coupled folding enables pathways inaccessible during equilibrium refolding.” You have not proven this with these simulations, so this assertion is not “established.” You can change the language here to “These results suggest more work should be done to see if CTF pathways are accessible during FF.” You have observed that the FF pathways do not sample the pathways observed in the CTF simulations – but you have not proven that it is not possible. You could seed the FF simulations with an intermediate to test the CTF pathway, you could find a reaction coordinate and make an argument energetically, you could search the other trajectories. More work needs to be done to “establish” it, especially with the limited number of paths that the current sampling returned.

**Response:** Thank you for your constructive comments. Indeed, the first reviewer also raised this issue. To address it, we increased the duration of the FF simulation trajectories (60 trajectories, each extended by 1 us) and the number of CTF simulation trajectories (adding two independent trajectories per transcription speed). Based on the updated simulation data, we identified a FF trajectory that successfully folded along PATH II. Therefore, our conclusion has been revised to state that FF exhibits a preference for PATH I, while CTF favors PATH II. In the revised manuscript, we have updated the previous results and discussion accordingly.

Figure 5: It is unclear what data you are incorporating into your conformational distribution plots. I think it would be great to have one set of plots where you include ALL simulated data, and one set of plots where you exclude all trajectories that did not fold. Also, please include an explanation for the arrows in the caption of this Figure.

**Response:** Thank you for your constructive suggestions. The original Fig. 5 used all simulated data. In the revised version, the new Fig. 5 incorporates data from successfully folded trajectories and now includes the results of CTF-1 and CTF-10, while the conformational distribution using all simulated data appears in Supplementary Fig. S11. As shown in the new Fig. 5, CTF conformations sampled across all three transcription speeds populate regions of smaller RMSD and RoG compared to FF. Since the intermediates of PATH II are inherently more compact than those of PATH I, these regions likely represent PATH-II intermediates, thereby rationalizing the preferential folding of CTF along PATH II. The same conclusion also holds when all trajectories are included (Supplementary Fig. S11). Additionally, the arrows in the original figure merely indicated the path from the intermediate state to the folded state but did not represent actual transition pathways. To avoid ambiguity, we have removed these arrows in the revised version.

**Fig. 5. Conformational distributions in 1ZIH simulations.** (a) Conformational distribution in free folding (FF) simulations. The reaction coordinates for the conformational space are the root-mean-square deviation (RMSD) of the stem region and the radius of gyration (RoG) of the entire 1ZIH molecule. (b-d) Conformational distribution in co-transcriptional folding (CTF) simulations at a transcription speed of 1, 10 and 100 ns/nt. The conformational distributions are built only on trajectory data from successful folding.

**Supplementary Fig. S11. Conformational distributions in 1ZIH simulations.** (a) Conformational distribution in free folding (FF) simulations. The reaction coordinates for the conformational space are the root-mean-square deviation (RMSD) of the stem region and the radius of gyration (RoG) of the entire 1ZIH molecule. (b-d) Conformational distribution in co-transcriptional folding (CTF) simulations at a transcription speed of 1, 10 and 100 ns/nt. The conformational distributions are built on all simulated trajectories.

Page 15, line 17-19: “In CTF simulations, in addition to the pathway present in FF simulations, we observed a folding pathway that do not exist in FF, where the pair distant from the loop form first, and the 4C-9G pair forms last.” This statement is only partially true – the h-bond vs. time plots in the SI Fig. 5 show that in multiple FF simulations, h-bonds form in the base pairs further away from the loop (yellow, red and green). The full TL never “folds,” but I’m not sure you’re adequately addressing all of the sampled data here. It might be interesting to understand what happens in the FF simulations when far h-bonds form, but the loop ultimately does not “fold.” It could certainly explain differences between each (FF and CTF) pathway.

**Response:** Thank you for pointing out this incorrect statement. It is true that in the FF simulations, some trajectories formed pairs distant from the loop, but they did not ultimately fold into the correct native structure. However, analyzing the reasons is challenging because our simulation time was insufficient to definitively determine whether successful folding could ultimately occur. For example, after extending the FF simulation time by 1 μs, we found that one trajectory followed PATH II, indicating initial formation of distant pairs prior to complete folding. Therefore, we consider this issue an open question for future exploration.

Page 16, line 3-5: “Finally, we propose a possible explanation for the mechanism by which CTF modifies folding pathways: viewing CTF as a form of FF that may possess rare initial conformations, which are difficult to sample in FF and direct RNA towards different folding pathways.” This is testable from the simulations you have run – why not look at the distribution of “starting” structures and compare using a combined clustering analysis or 2d RMS? You can also test whether any of the FF simulations sample any of the CTF structures by comparing all structures with a 2D RMS plot. You could also measure backbone dihedrals to ascertain whether structures in the CTF are not seen in the FF simulations.

**Response:** Thank you for your insightful suggestion. To maintain consistency with the previous analysis, we retained RMSD and RoG as coordinates for conformational distribution. Fig. 6a displays the conformational distributions at T_F_ = 0 ns (the free folding time, defined as the time elapsed after transcription completion) for all trajectories. Crucially, slower transcription yields more pronounced differences between CTF and FF distributions at T_F_ = 0 ns. Tracking conformational evolution at later timepoints (T_F_ = 10, 100, and 1000 ns; Figs. 6b-d), we observe that while differences between FF and CTF distributions diminish over time, the discrepancy still persists. These results elucidate a pathway regulation mechanism for CTF: Transcription generates compact initial conformations rarely sampled in FF. Consequently, CTF establishes a distinct preference for folding pathways during the subsequent post-transcriptional folding compared to FF, and this pathway preference is modulated by transcription speed.

This mechanism finds compelling parallel in protein biosynthesis: Till et al. (PNAS 122, e2422678122, 2025) demonstrated that the ribosome and Trigger Factor collaboratively compact nascent proteins to accelerate folding. Despite differences between biological systems (protein vs. RNA), these analogous findings strongly support our proposed pathway regulation mechanism in CTF.

**Fig. 6. Conformational evolution of 1ZIH.** (a–d) Conformational distributions of 1ZIH under four simulation scenarios (FF, CTF-1, CTF-10, and CTF-100) at different free-folding times (T_F_ = 0, 10, 100, and 1000 ns). In FF simulations T_F_ equals the actual simulation time, whereas in CTF simulations T_F_ is obtained by subtracting the transcription duration (11Δt) from the actual simulation time. The reaction coordinates describing the conformational space are the root-mean-square deviation (RMSD) of the stem region and the radius of gyration (RoG) of the entire 1ZIH molecule. Small semi-transparent symbols denote the conformations of 1ZIH at T_F_; red squares, green triangles, blue stars, and purple circles correspond to the FF, CTF-1, CTF-10, and CTF-100 simulations, respectively. Large opaque symbols represent the means of each group, with error bars indicating the standard deviations.

**-----------------**

**Reviewer #3**

**-----------------**

In this manuscript, the authors applied all-atom MD simulation to study the effect of transcription on the folding pathways of an RNA hairpin. They implement a movable rigid plane to prevent non-physical interactions between synthesized and unsynthesized nucleotides and obtained that RNA transcription may diversify folding dynamics. However, the manuscript needs minor revision to improve the readability.

**Response:** Thank you for highlighting the need to improve readability. In this revision we have thoroughly edited the entire manuscript according to your comments. We hope these changes make the manuscript substantially easier to read and they meet with your approval.

1) Page 7, the initial structure should be presented, is Fig1b the intial structure?

**Response:** Yes, Fig. 1b explicitly shows the initial structure used in all simulations. This extended conformation was generated using the *tleap* program in Amber18 package. We have clarified this in the Methods section of the revised manuscript.

2) Page 6, is the periodic boundary conditions used? If it is, as the initial structure is extended in x-direction, what is thickness of solvent layer at each direction?

**Response:** Yes, periodic boundary conditions were applied in all simulations. In our study, the water box dimensions with a 12 Å buffer are [81.1 Å × 47.1 Å × 56.2 Å]. We have added this description in the Methods section of the revised manuscript.

3) Fig5, is the distribution of one pathway or all simulations? If it is of one pathway, as there are 3 pathways for FF folding, is the distribution same for each pathway? and there are two kinds of pathway for CFT folding, Fig5b represents which pathway?

**Response:** We apologize for the unclear presentation. The original Fig. 5 used all simulated data. In the revised version, the new Fig. 5 incorporates data from successfully folded trajectories and now includes the results of CTF-1 and CTF-10, while the conformational distribution using all simulated data appears in Supplementary Fig. S11. As shown in the new Fig. 5, CTF conformations sampled across all three transcription speeds populate regions of smaller RMSD and RoG compared to FF. Since the intermediates of PATH II are inherently more compact than those of PATH I, these regions likely represent PATH-II intermediates, thereby rationalizing the preferential folding of CTF along PATH II. The same conclusion also holds when all trajectories are included (Supplementary Fig. S11).

**Fig. 5. Conformational distributions in 1ZIH simulations.** (a) Conformational distribution in free folding (FF) simulations. The reaction coordinates for the conformational space are the root-mean-square deviation (RMSD) of the stem region and the radius of gyration (RoG) of the entire 1ZIH molecule. (b-d) Conformational distribution in co-transcriptional folding (CTF) simulations at a transcription speed of 1, 10 and 100 ns/nt. The conformational distributions are built only on trajectory data from successful folding.

**Supplementary Fig. S11. Conformational distributions in 1ZIH simulations.** (a) Conformational distribution in free folding (FF) simulations. The reaction coordinates for the conformational space are the root-mean-square deviation (RMSD) of the stem region and the radius of gyration (RoG) of the entire 1ZIH molecule. (b-d) Conformational distribution in co-transcriptional folding (CTF) simulations at a transcription speed of 1, 10 and 100 ns/nt. The conformational distributions are built on all simulated trajectories.

4) FigS9, are the hydrogen bonds of the non-native base pair in the structure of the intermediate state formed? Could the structure be found in the FF simulations except the 3 successful folding simulations?

**Response:** Yes, the intermediate state in Supplementary Fig. S9 features two adjacent non-native base pairs, which subsequently rearrange into native pairs during folding. This specific intermediate was not observed in any FF trajectory; nevertheless, non-native-to-native transitions of base pairs do occur in FF folding, as illustrated in Supplementary Fig. S13.

Supplementary Fig. S13. An example of the trajectory of non-native-to-native base pair transitions in FF simulations.
